# Deciphering the Pharmacological Mechanisms of Qidan Dihuang Decoction in Ameliorating Renal Fibrosis in Diabetic Nephropathy through Experimental Validation *In Vitro* and *In Vivo*

**DOI:** 10.1155/2022/4137578

**Published:** 2022-08-10

**Authors:** Qiuer Liang, Zhenyu Bai, Ting Xie, Hanqi Lu, Lei Xiang, Ke Ma, Tianhao Liu, Tingting Guo, Liguo Chen, Xiaoshan Zhao, Ya Xiao

**Affiliations:** ^1^School of Traditional Chinese Medicine, Jinan University, Guangzhou, China; ^2^Affiliated Dongguan People's Hospital, Southern Medical University (Dongguan People's Hospital), Dongguan, China; ^3^Department of Nephrology, Dongguan Traditional Chinese Medicine Hospital, Dongguan, China; ^4^Department of Integrative Chinese and Western Medicine, The First Affiliated Hospital of Guangdong Pharmaceutical University, Guangzhou, China; ^5^Department of Nephrology, Integrated Hospital of Traditional Chinese Medicine, Southern Medical University, Guangzhou, China; ^6^Department of Gastroenterology, Affiliated Hospital of Jiangnan University, Wuxi, Jiangsu, China; ^7^Wuxi School of Medicine, Jiangnan University, Wuxi, Jiangsu, China; ^8^Department of Nephrology, Zhujiang Hospital of Southern Medical University, Guangzhou, China; ^9^School of Traditional Chinese Medicine, Southern Medical University, Guangzhou, China

## Abstract

**Objective:**

QiDan DiHuang decoction (QDD) has been proven to have good efficacy in decreasing albuminuria levels, improving renal function, and inhibiting renal fibrosis in diabetic nephropathy (DN). However, the potential mechanism remains unclear. The purpose of this study was to explore the underlying mechanism of QDD for treating DN *in vitro* and *in vivo*.

**Methods:**

Db/db mice were treated with QDD or saline intragastrically for 12 weeks. Non-diabetic db/m mice were used as controls. Rat renal tubular epithelial cells (NRK-52E) were cultured in high glucose conditions. ATF4 siRNA was transfected into NRK-52E cells. Different indicators were detected via UPLC, RT-PCR, western blotting, cell viability assays and apoptosis, transmission electron microscopy, histology, and immunofluorescence staining.

**Results:**

Db/db mice experienced severe kidney damage and fibrosis, increased levels of PERK, eIF2*α*, and ATF4, and suppression of renal autophagy compared with db/m mice. The results showed a significant improvement in glucose intolerance, blood urea nitrogen, urine albumin, serum creatinine, and renal fibrosis in db/db mice with QDD treatment. Meanwhile, the application of QDD resulted in the downregulation of PERK, eIF2*α,* and ATF4 and the upregulation of autophagy in diabetic kidneys. *In vitro*, the exposure of NRK-52E cells to high glucose resulted in downregulation of the ratio of LC3-II/LC3-I and upregulation of P62, a reduction in the number of autophagosomes and upregulation of fibronectin (FN), collagen IV and TGF-*β*1 protein, which was reversed by QDD treatment through inhibiting ATF4 expression.

**Conclusions:**

Taken together, our results suggest that QDD effectively alleviates diabetic renal injuries and fibrosis by inhibiting the PERK-eIF2*α*-ATF4 pathway and promoting autophagy in diabetic nephropathy.

## 1. Introduction

Diabetic nephropathy (DN) is known to be a major complication of diabetes and is also the main cause leading to kidney failure [1]. The pathogenesis of DN is complicated and multifactorial. The major pathological changes of DN manifest as mesangial expansion, thickened basement membrane, and renal fibrosis. Renal fibrosis is an important factor for kidney function decline in DN and progression to ESRD, characterized by excessive deposition of extracellular matrix components, such as collagens and fibronectin [2, 3]. Therefore, elucidating the mechanism of renal fibrosis and developing effective treatment methods are urgent problems to be solved.

Traditional Chinese medicine (TCM) has significant advantages and has been beneficial in treating diabetes and its complications for hundreds of years, especially in the prevention and treatment of DN [4, 5]. Qidan Dihuang decoction (QDD) is an herb compound for the treatment of DN, which was developed via literature research and data analysis, and has been patented (Patent No. ZL201310015464.5). QDD is composed of *Astragalus membranaceus* (Huang Qi), *Salvia miltiorrhiza Bunge* (Dan Shen), *Rehmannia glutinosa* (Gaertn) (Di Huang), *Dioscoreae rhizoma* (Shan Yao), and *Glycyrrhiza uralensis* (Gan Cao). Our previous study reported that QDD has remarkable clinical efficacy in improving albuminuria levels in patients with DN [6]. In addition, QDD has a protective effect on the kidneys of diabetic rats and it can reverse the inflammatory milieu of DN serum [7]. We also found that QDD can significantly reduce serum creatinine and 24 h proteinuria in rats with DN and improve the levels of fibrosis marker proteins SMA-*α* and TGF-*β* [8]. Therefore, QDD is considered a good candidate for preventing renal fibrosis in DN. However, the protective mechanism of QDD against DN is unknown.

Autophagy is a lysosomal degradation pathway. The activation of autophagy can promote the degradation of protein aggregates and damaged organelles, thereby helping maintain intracellular homeostasis [9]. Multiple studies have confirmed that inhibition of autophagy plays a pivotal role in the pathogenesis of diabetic nephropathy. Activation of autophagy is beneficial for improving kidney injury and restoring renal function. Therefore, improving autophagy has become an important therapeutic target in DN treatment [10, 11]. Autophagy activation can be regulated by multiple pathways, including the mechanistic target of the rapamycin (mTOR) pathway and the AMP-activated protein kinase (AMPK) pathway. However, studies have suggested that endoplasmic reticulum stress (ERS) is also a key way to regulate autophagy activity [12]. Moreover, ERS has been increasingly recognized as a critical mechanism in the pathogenesis of DN [13, 14]. The unfolded protein response (UPR), mediated by endoplasmic reticulum stress, is designed to eliminate the accumulation of misfolded proteins [15]. The three main arms of the UPR are mediated by protein kinase RNA (PKR)-like ER kinase (PERK), inositol-requiring 1 (IRE1), and activating transcription factor 6 (ATF6). Studies suggested that the PERK/eIF2*α*/ATF4 pathway was involved in the regulation of autophagy [16]. Our previous study showed that the ERS-inducible transcription factor ATF4 promotes tubulointerstitial fibrosis by inhibiting autophagy in DN [17]. Therefore, we speculate that the mechanism of QDD delaying renal fibrosis in DN is related to the regulation of autophagy via the PERK/eIF2*α*/ATF4 signaling pathway.

This study aimed to explore the effect of QDD in improving renal fibrosis in DN and the effect of QDD in regulating the PERK/eIF2*α*/ATF4 signaling pathway and renal autophagy. We demonstrated that QDD suppressed renal fibrosis in DN by inhibiting the PERK/eIF2*α*/ATF4 signaling pathway and restoring autophagy activity.

## 2. Materials and Methods

### 2.1. QDD Preparation and UPLC-QTOF-MS Analysis of QDD

QDD is comprised of five herbs: 30 g of *Astragalus membranaceus*, 15 g of *Rehmannia glutinosa* (Gaertn), 15 g of *Dioscoreae rhizoma*, 15 g of *Salvia miltiorrhiza bunge*, and 5 g of *Glycyrrhiza uralensis*. The herbs were soaked in 500 ml distilled water for 30 minutes and boiled twice for one hour per time. After filtering with sterile gauze, the extraction liquid was centrifuged, and the supernatant was dried in a vacuum freeze dryer and stored at −80°C.

About 100 mg sample was added into 500 *μ*L extract, homogenized for 4 minutes, placed in an ice-water bath, sonicated for 1 hour, and then allowed to stay at −20°C for 1 hour. The sample was centrifuged at 12000 rpm for 10 minutes at 4°C. Then, 200 *μ*l of supernatant was taken for the test. The target compound was analyzed by UPLC-QTOF-MS, and 0.1% formic acid (solvent A) and acetonitrile 0.1% formic acid (solvent B) were used for gradient elution. Linear gradient elution was conducted as follows: 0–3.5 minutes at 5–15% B, 3.5 to 6.5 minutes at 15–30% B, 6.5 to 12.5 minutes at 30–70% B, 12.5 to 18 minutes at 70–100% B, and 18 to 22 minutes at 100% B. In addition, based on the response value, methylnissolin 3-O-glucoside, trifolirhizin, berberrubine, and prazewaquinone A were selected for quantitative analysis.

### 2.2. Animal Model and Treatments

C57BL/KsJ leptin receptor-deficient (db/db) and db/m Six-week-old male mice were purchased from the Model Animal Research Center of Nanjing University. Both db/db and db/m mice were raised at 21 ± 3°C in a 12-hour light/dark cycle environment. Before the experiment, all experimental mice were adaptively fed for 1 week, and they had free access to food and water. Six db/m mice were used as controls. According to the random number table method, 12 db/db mice were divided into the db/db group and db/db + QDD group, with six mice in each group. Mice in the db/db + QDD group were given QDD by gavage at 10 g/kg/day, whereas the mice in the db/db group and db/m group were administered saline. The body weight and blood glucose of the mice were measured once a week, and basic data such as the health status, food intake, and water consumption of the mice were observed and recorded. Twelve weeks later, the mice were moved to the euthanasia workstation and anesthetized with sodium pentobarbital (60 mg/kg iP). After the collection of blood samples from the orbital venous, the renal tissues of the mice from each group were immediately collected according to the different testing protocols. The blood glucose test in this experiment was performed with a blood glucose meter (Roche Diabetes Care GmbH, UK). The animal experiments were approved by the Animal Experiment Ethics Committee of Jinan University (approval number: 2019022501).

### 2.3. Cells and Transfection

The rat renal tubular epithelial cells (NRK-52E cells) were obtained from the Cell Bank of the Chinese Academy of Sciences. A medium containing 5% fetal bovine serum (Gibco), 94% DMEM (Gibco), and 1% penicillin-streptomycin (Gibco) was used for cell culture. The cell culture environment was 37°C, containing 5% carbon dioxide. NRK-52E cells were treated with 5.5 mmol/L D‐glucose (normal glucose, NG group), 30 mmol/L D‐glucose (high glucose, HG group) for 48 hours or 30 mmol/L D‐glucose plus QDD at 200 *μ*g/mL, 1200 *μ*g/mL and 2000 *μ*g/mL concentrations. The ATF4 siRNA and siRNA-NC vectors were synthesized by Guangzhou Ruibo Biological Co., Ltd. (Guangdong, China). All cell transfection experiments used Lipofectamine 2000 transfection reagent (Invitrogen, Carlsbad, CA, USA) and OPTI-MEM medium (Gibco).

### 2.4. Western Blotting

Proteins were extracted from kidney tissue samples and NRK-52E cells using the RIPA method. Protein concentrations were measured by a BCA protein detection kit. After SDS-PAGE gel fractionation, membrane transfer and blot blocking, the membrane was incubated with the following primary antibodies overnight: anti-collagen type 4 (Col-IV) (1 : 2000, NB120-6586SS), anti-LC3A/B (1 : 500, 12741S), anti-P62 (1 : 1000, 23214S), anti-ATF4 (1 : 1000, 11815S), anti-GAPDH (1 : 10000, KC-5G5), anti-fibronectin (FN) (1 : 5000, ab45688), and anti-TGF-*β*1 (1 : 1000, ab92486). Then the membrane was incubated with the secondary antibodies: goat anti-rabbit IgG (H + L), mouse/human ads-HRP (1 : 20000, 4050-05), and rabbit anti-mouse IgG (H + L)-HRP (1 : 10000, 6170-05). Immunoblots were performed with enhanced chemiluminescence (ECL). Densitometry analysis of the bands was performed by Gel-pro analyzer software.

### 2.5. Oral Glucose Tolerance Test (OGTT)

Mice were fasted for 6 h and then given glucose solution by gavage at 2.0 g/kg. Then, at 0, 30, 60, 90, and 120 minutes, blood was drawn from the tail vein, and the blood glucose at each time was measured with a blood glucose meter. Finally, the blood glucose curve was drawn and its area under the curve (AUC_PG_) was calculated. Area of blood glucose curve (AUC_PG_) = 15 × FPG + 30 × PG_30_ + 30 × PG_60_ + 30 × PG_90_  + 15 × PG_120._

### 2.6. Biochemical Analysis

Serum creatinine (Scr) and blood urea nitrogen (BUN) were measured by an automatic chemical analyzer (AU480; Beckman Coulter Inc). A blood glucose meter (Roche Diabetes Care GmbH, UK) was used to detect blood glucose. In addition, mouse metabolic cages were used to collect mouse urine for 24 hours, and urinary albumin was detected by ELISA.

### 2.7. Quantitative Real-Time PCR Analysis

The total RNA was extracted from the samples using TRIzol kit (Invitrogen, Carlsbad, CA). Then, reverse transcription was performed using an Invitrogen (Carlsbad, CA) kit. Real-time quantitative RT-PCR was performed by the Bio-Rad 96FX circulation system (Bio-Rad, USA) with SYBR Green Master Mix. The primer sequences of the related target gene and GAPDH reference gene were as follows: ATF4: forward 5′-CGACTTTTATTACACTTTCTGGGAG-3′ and reverse 5′-GGGACAGATTGGATGTTGGAG-3′; eIF2*α*: forward 5′-AGCATTCTTCGCCATGTTGC-3′ and reverse 5′-TAGGCACCGTATCCAGGTCT-3′; PERK: forward 5′-CCGCAAGAAGGACCCTATCC-3′ and reverse 5′-GAGTTTCAGACTCCTTCCGCT-3′; GAPDH: forward 5′-TCTCTGCTCCTCCCTGTTC-3′ and reverse 5′-ACACCGACCTTCACCATCT-3′. The results were analyzed by the 2^−ΔΔCq^ method.

### 2.8. Cell Viability Assay

NRK-52E cells (5 × 10^3^ cells/well) were seeded into 96-well plates and treated with high glucose for 48 hours or QDD for 12 hours. The viability was evaluated with a Cell Counting Kit 8 (Dojindo Laboratories, Kyushu, Japan).

### 2.9. Cell Apoptosis

The cells were trypsinized without EDTA and collected. The cells were washed twice with PBS, and the supernatant was discarded after centrifugation. The experiment was performed according to the instructions of the Annexin-V-FITC detection kit (K201-100, BioVision, USA). In short, 100 *μ*L of binding buffer was added to prepare the cell suspension. Then, 2.5 *μ*L of Annexin V-FITC and 5 *μ*L of propidium iodide (PI) were added to the cell suspension. All samples were incubated in a dark environment at room temperature for 15 minutes, and 200 *μ*l binding buffer was gently mixed with the cells. Flow cytometry was used to detect the level of cell apoptosis (version 10.0, FlowJo, FACS CaliburTM, BD, USA).

### 2.10. Histopathology and Immunofluorescence

The kidney was quickly removed and washed with physiological saline, fixed in 4% paraformaldehyde for 30 minutes, and dehydrated. Finally, it was infiltrated by xylene and embedded in paraffin. The samples were cut into 5 *μ*m thick paraffin sections. Following conventional staining steps, the sections were stained with HE, Periodic acid-Schiff (PAS), and Masson's trichrome. The mesangial expansion index was measured to evaluate the quantifications of PAS staining. Positive signals were quantified using Image-Pro Plus 6.0 software (Media Cybernetics, Bethesda, MD) in six randomly selected visual fields at 200× magnification. The mesangial expansion index was expressed as a percentage positive area of the entire glomerulus. Collagen fibrils area ratio was measured to evaluate the quantifications of Masson staining. The collagen deposition in the kidney was assessed by Image-Pro Plus 6.0 software (Media Cybernetics, Bethesda, MD). For each experimental group, six fields were randomly selected at 200× magnification. Collagen fibrils area ratio was expressed as a percentage of blue fibrotic area relative to the entire area.

Immunofluorescence staining was used to detect the expression of renal light chain 3 (LC3B). After antigen retrieval and blocking, the kidney tissue sections were incubated with anti-LC3B (1 : 200, CST) at 4°C for 12 hours. After returning to room temperature, the samples were washed with PBS and incubated with ALexa Fluor 594-conjugated goat anti-rabbit IgG, FITC-conjugated goat anti-mouse IgG, and cy3-conjugated goat anti-rabbit IgG at 37°C for 1 hour. DAPI under dark conditions was used to label the cell nucleus, and the sample was washed with PBS. A laser scanning confocal fluorescence microscope was used to observe the sections and collect images (UltraVIEW VoX; PerkinElmer, Inc). The fluorescence activity was measured to evaluate the quantifications of immunofluorescence staining. The fluorescence activity of LC3B was expressed as the percentage integrated optical density of the entire area. For each experimental group, six fields were randomly selected and the mean fluorescence activity was calculated.

### 2.11. Transmission Electron Microscopy

After the experiment, the cells were collected, and the samples were immediately fixed in 2.5% glutaraldehyde. The samples were rinsed 4 times with 0.1 mol/L phosphate buffer and then fixed with 1% osmium tetroxide at 4°C. After dehydration, the samples were embedded in Durcupan ACM. Finally, the samples were stained with uranyl acetate and lead citrate and sectioned. A transmission electron microscope was used to observe the autophagosomes (JEOL-100CXII, JEOL, Japan). Ten fields of view from each group were selected randomly for observation.

### 2.12. Statistical Analysis

The data are expressed as the mean ± SE, and SPSS software 22.0 (SPSS, Inc) was used for statistical analysis. The student's *t*-test was applied to determine significant differences between two independent groups. For multiple groups, one-way ANOVA with Tukey's post hoc test was used. *P* < 0.05 was considered statistically significant.

## 3. Results

### 3.1. Chemical Profile of QDD by UPLC-QTOF-MS

**Figure 1:**
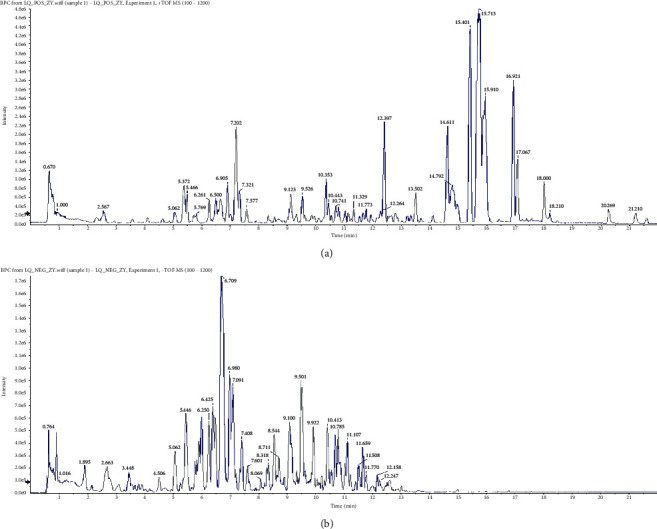
Base peak intensity of QDD in (a) positive and (b) negative ion modes by UHPLC-QTOF-MS.

**Figure 2:**
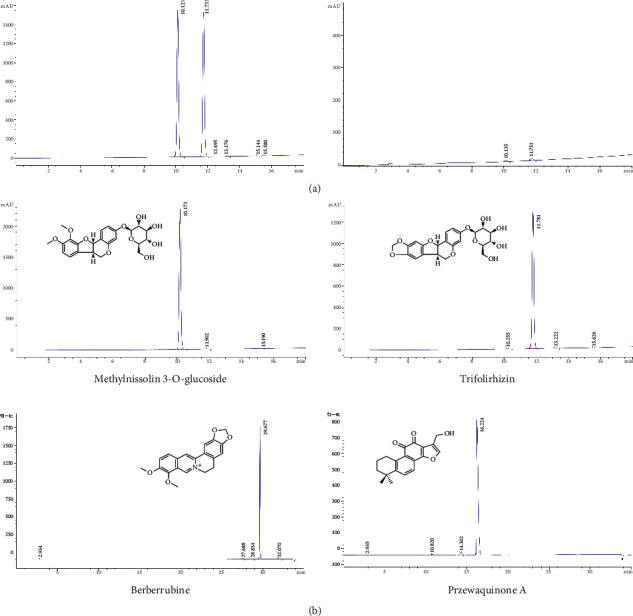
Quantitative analysis of potential compounds in QDD by UPLC-Q/TOF-MS. (a) Representative peak chromatograms of qualitative mixed standards. (b) The representative peaks of compounds identified in QDD include methylnissolin 3-O-glucoside, trifolirhizin, berrubine and prazewaquinone A.

The UPLC-Q-TOF/MS method was developed for the qualitative analysis of QDD. The top 10 chemical components identified from QDD based on the base peak intensity included methylnissolin 3-O-glucoside, trifolirhizin, berberrubine, przewaquinone A, bractealine, DL-pyroglutamic acidDL, astragaloside VII, isomucronulator 7-O-glucoside, 3,4-dihydroxybenzaldehyde3,4, naringenin chalcone, yunnaneic acid G, danshenspiroketallactone, stachyose, guanosine, licochalcone B, 1,3,7-trihydroxyxanthone1,3,7, gancaonin O, isoanhydroicaritin, 24-methylene cholesterol and blepharolide B, etc (Table 1). The chromatogram determined by UPLC-Q/TOF-MS showed the chemical basis peak intensity in positive and negative ion modes for the chemical components of QDD (Figure 1). We used UPLC-Q/TOF-MS to quantitatively evaluate the potential compounds, including methylnissolin 3-O-glucoside, trifolirhizin, berberrubine, and prazewaquinone A (Figure 2). The results showed that QDD contained 0.98080 *μ*g/mL methylnissolin 3-O-glucoside, 0.28191 *μ*g/mL trifolirhizin, 37.56526 *μ*g/mL berberrubine, and 300.07802 *μ*g/mL prazewaquinone A. The four pharmacological ingredients are listed in Table 2.

### 3.2. QDD Improved Kidney Injury in Db/Db Mice

**Figure 3:**
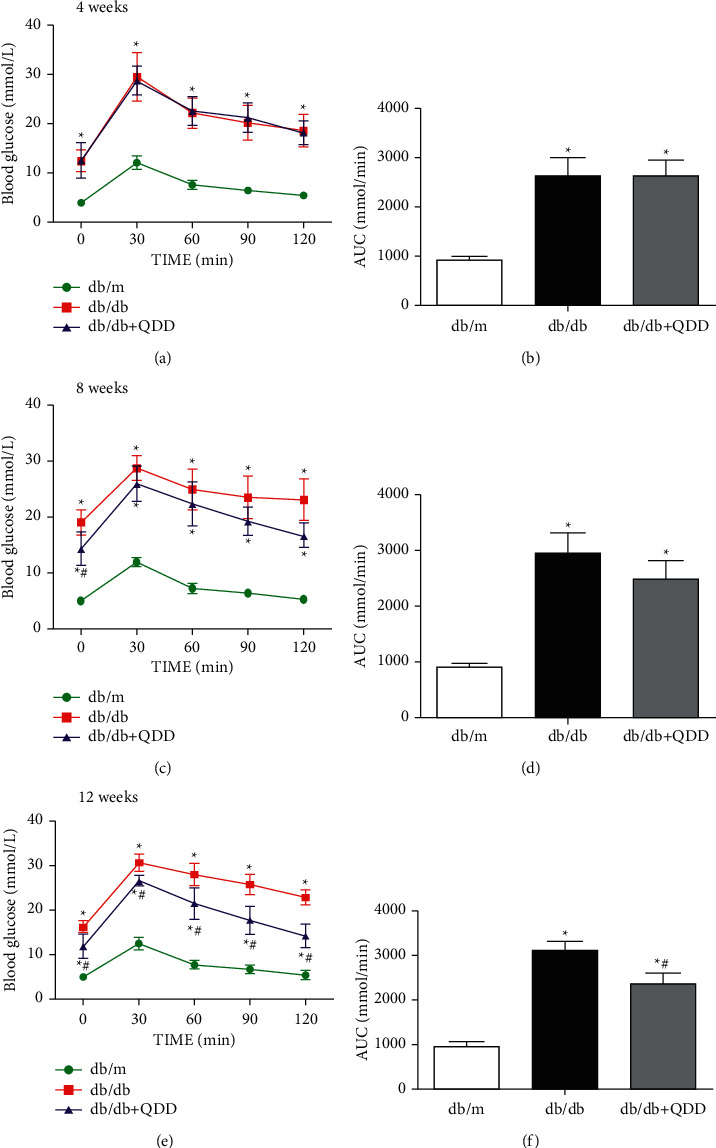
Effects of QDD on OGTT and AUC in db/db mice. (a), (c), (e) The levels of OGTT (*n* = 6) were detected by a glucometer. (b), (d), (f) Based on the OGTT results, the AUC was calculated (*n* = 6). Data are presented as the mean ± SD values. ^*∗*^*P* < 0.05 vs. db/m; ^#^*P* < 0.05 vs. db/db. AUC, Area under the curve; OGTT, Oral glucose tolerance test.

**Figure 4:**
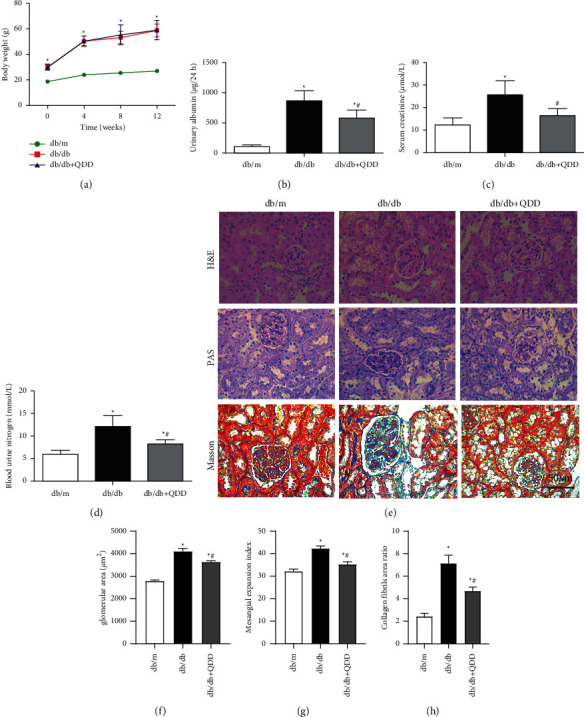
QDD improves kidney damage in db/db mice. (a) The body weights of mice were recorded every 4 weeks from 0 weeks to 12 weeks (*n* = 6). (b) The levels of urinary albumin (*n* = 6) were detected by an automatic biochemistry analyser. (c) The levels of serum creatinine (*n* = 6) were detected by an automatic biochemistry analyser. (d) The levels of blood urea nitrogen (*n* = 6) were detected by an automatic biochemistry analyser. (e) The pathologic changes in renal tissues were observed via HE, PAS and masson staining. (f) Glomerular area. (g, h) Quantifications of PAS and masson staining. Data are presented as the mean ± SD values. ^*∗*^*P* < 0.05 vs. db/m; ^#^*P* < 0.05 vs. db/db.

To evaluate the effects of QDD on the renal function of db/db mice, body weight, urinary albumin urea nitrogen, serum creatinine, and glucose tolerance were measured in each group. The results suggested that db/db mice showed obvious glucose intolerance compared with db/m mice. The glucose intolerance of the QDD group was significantly improved (Figures 3(a)–3(f)). Compared to the db/m group, the body weight of the db/db group and QDD group were obviously increased (Figure 4(a)). In addition, the urinary albumin, urea nitrogen, and creatinine levels of the db/db group were obviously increased compared with the db/db group. The QDD group showed significantly reduced urinary albumin, urea nitrogen, and creatinine compared with the db/db group (Figures 4(b)–4(d)). We used HE, PAS, and Masson staining methods to detect pathological changes in the kidney tissue. Mesangial matrix and glomerular volume expansion, mesangial cell proliferation, thickening of the basement membrane, and evident renal fiber accumulation were observed in the kidney tissue of the db/db mice. Compared to with the db/db group, QDD treatment alleviated these pathological injuries (Figures 4(e)–4(h)). According to the results above, QDD improved glucose intolerance, renal function, urinary albumin, and pathological injuries.

### 3.3. QDD Ameliorated Renal Fibrosis and Inhibited the PERK-eIF2*α*-ATF4 Pathway in Db/Db Mice

**Figure 5:**
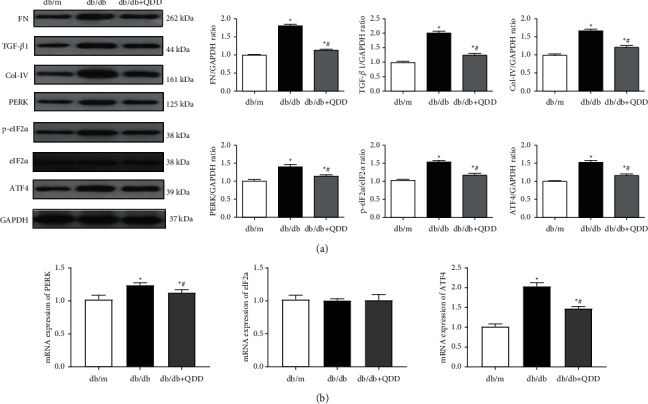
QDD ameliorates kidney fibrosis and inhibited the PERK-eIF2*α*-ATF4 pathway in db/db mice. (a) The expression and densitometry analysis of FN, TGF-*β*1, collagen type 4 (Col-IV), PERK, eIF2*α*, p-eIF2*α* and ATF4 (*n* = 3). (b) The mRNA levels of PERK, eIF2*α*, and ATF4 in renal tissue were determined by qRT-PCR (*n* = 6). Data are presented as the mean ± SD values. ^*∗*^*P* < 0.05 vs. db/m; ^#^*P* < 0.05 vs. db/db.

To clarify the effects of QDD on renal fibrosis, the expression of FN, Col-IV, and TGF-*β*1 was evaluated by western blot. The western blot results revealed that the expression levels of FN, TGF-*β*1, and Col-IV increased significantly in the db/db group compared with the db/m group. QDD remarkably reduced FN, TGF-*β*1 and Col-IV expression in the kidney compared with the db/db group (Figure 5(a)). To further explore the potential mechanism of QDD in the treatment of DN, we tested the expression of PERK, eIF2*α*, and ATF4, which are related to endoplasmic reticulum stress. The results showed that the mRNA expression of PERK, ATF4 and protein expression of PERK, p-eIF2*α* and ATF4 in the kidney tissue of db/db mice were markedly increased, while the mRNA level and total protein expression of eIF2*α* was not changed in the db/db mice. After QDD treatment, the mRNA expression of PERK, ATF4 and protein expression of PERK, p-eIF2*α* and ATF4 showed a significant decline (Figures 5(a) and 5(b)). Protein phosphorylation is an important mechanism to regulate and control protein activity and function. Our results suggested that protein eIF2*α* had high activity rather than total amount changes and QDD has effects on phosphorylation of eIF2*α*. These results indicated that QDD can improve renal fibrosis and inhibit the PERK-eIF2*α*-ATF4 pathway in db/db mice.

### 3.4. QDD Promoted Autophagy in Db/Db Mice

**Figure 6:**
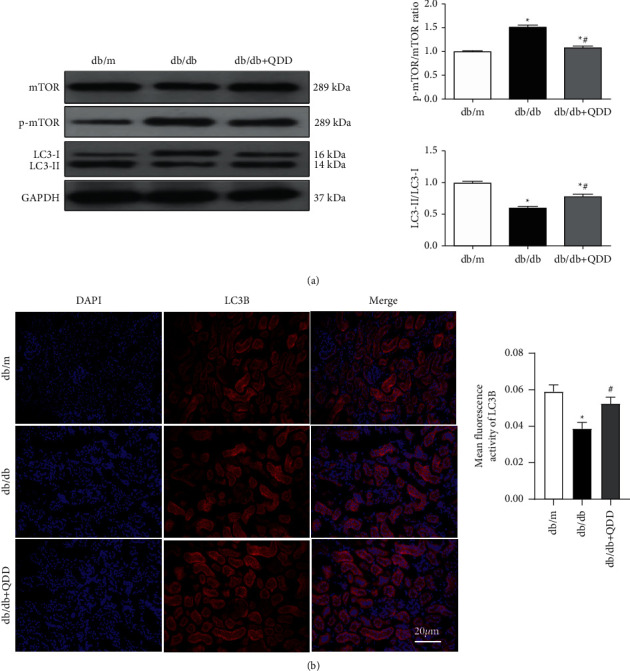
The effect of QDD on autophagy-related proteins in the kidneys of db/db mice. (a) The expression and densitometry analysis of mTOR, p-mTOR, and LC3II/LC3I (*n* = 3). (b) The expression of LC3B was determined via immunofluorescence. The fluorescence intensity of LC3B was analyzed by Image J software. The nucleus staining is shown in blue, LCB staining is shown in red, the bright red spot is the LCB fluorescent spot (white arrows), scale bar = 20 *μ*m. Data are presented as the mean ± SD values. ^*∗*^*P* < 0.05 vs. db/m; ^#^*P* < 0.05 vs. db/db.

We detected the expression of the autophagy-related proteins mTOR, p-mTOR, and LC3 in db/db mice. The expression of p-mTOR in the kidneys of db/db mice significantly increased, and the ratio of LC3-II/LC3-I markedly decreased compared with that in db/dm mice. After QDD treatment, db/db mice showed downregulation of p-mTOR and upregulation of the ratio of LC3-II/LC3-I (Figure 6(a)). In addition, the results of immunofluorescence also showed that the expression of LC3B in the kidneys of db/db mice was decreased compared with that in db/dm mice. QDD significantly increased the expression of LC3B (Figure 6(b)). Our results demonstrated that QDD can inhibit the PERK-eIF2*α*-ATF4 pathway and promote autophagy in db/db mice.

### 3.5. QDD Alleviated HG-Induced NRK-52E Cells Injury

**Figure 7:**
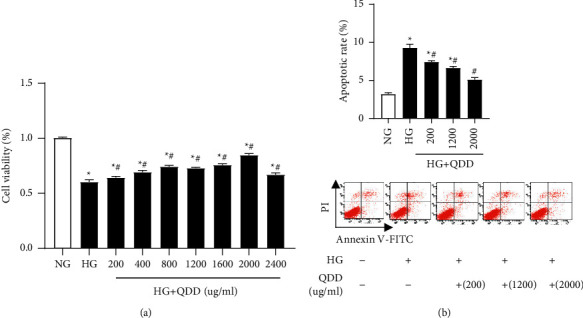
The effect of QDD on the viability and apoptosis of HG-treated NRK-52E cells. (a) Cell viability was detected by Cell Counting Kit-8. (b) Flow cytometry analysis was performed to evaluate cell apoptosis in the treated NRK-52E cells. Data are presented as the mean ± SD values. ^*∗*^*P* < 0.05 vs. NG; ^#^*P* < 0.05 vs. HG. NG: low glucose; HG: high glucose.

To determine whether QDD affected cell viability, we used a CCK-8 assay to assess the effect of QDD on the viability of NRK-52E cells. NRK-52E cells were treated with high glucose (HG) combined with QDD at various concentrations, ranging from 200 *μ*g/mL to 2400 *μ*g/mL. Treatment with QDD at concentrations from 200 *μ*g/mL to 2000 *μ*g/mL markedly improved the survival rate of the NRK-52E cells. However, a significant reduction in cell viability and survival rate was observed in the cells treated with 2400 *μ*g/ml QDD (Figure 7(a)). Based on these findings, QDD at 200 *μ*g/mL, 1200 *μ*g/mL, and 2000 *μ*g/mL concentrations were used in the subsequent experiments. Additionally, we also observed the effect of QDD on the apoptosis rate of the cells and found that QDD can significantly reduce the level of cell apoptosis (Figure 7(b)).

### 3.6. QDD Inhibited the Expression of ATF4 and Col-IV and Promoted Autophagy in HG-Treated NRK-52E Cells

**Figure 8:**
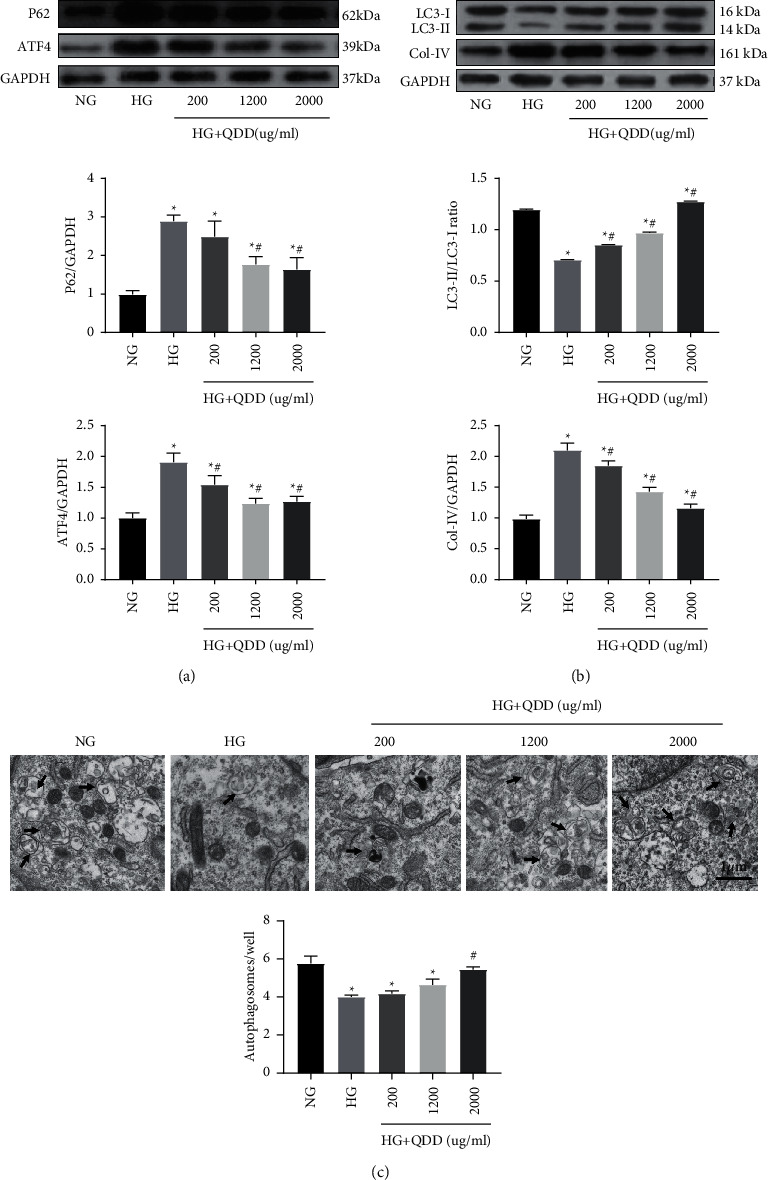
The effect of QDD on ATF4, fibrosis and autophagy in HG-treated NRK-52E cells. (a) Expression and densitometry analysis of P62 and ATF4 (*n* = 3). (b) The expression and densitometry analysis of LC3 and Col-IV (*n* = 3). (c) Representative electronic micrographs and summarized data showing the number of autophagosomes/well in different groups (autophagosomes were counted in 10 randomly selected fields). Autophagic vacuoles are indicated with arrows. Data are presented as the mean ± SD values. ^*∗*^*P* < 0.05 vs. NG; ^#^*P* < 0.05 vs. HG. NG: low glucose; HG: high glucose.

To clarify the effect of QDD on ATF4, fibrosis levels, and autophagy in HG-treated NRK-52E cells, we used western blotting to detect the expression of ATF4, Col-IV, and the autophagy-related proteins P62, LC3-I, and LC3-II. The results indicated that the protein expression of ATF4, P62, and fibrosis marker protein Col-IV was remarkably upregulated in HG-treated NRK-52E cells compared with cells cultured in NG conditions. At the same time, the ratio of LC3-II/LC3-I decreased markedly in HG-treated NRK-52E cells. The cells exposed to HG combined with 200 *μ*g/mL, 1200 *μ*g/mL, or 2000 *μ*g/mL QDD showed downregulation of ATF4 and Col-IV and upregulation of the ratio of LC3-II/LC3-I. However, HG-induced cells treated with 1200 *μ*g/mL or 2000 *μ*g/mL QDD showed a significantly reduced level of P62 (Figures 8(a) and 8(b)). Moreover, we also detected the changes in the autophagic vesicles in NRK-52E cells by transmission electron microscopy. In each group, 10 regions were randomly selected for observation, and the number of autophagosomes was calculated and counted. The results showed that compared with NRK-52E cells cultured in NG conditions, the number of autophagosomes in NRK-52E cells cultured in HG conditions was significantly reduced. The number of autophagosomes in the cells cultured under HG conditions with 2000 *μ*g/mL QDD was obviously increased compared with that in the cells cultured under HG conditions (Figure 8(c)). Our results verified that QDD inhibited the expression of ATF4 and Col-IV and promoted autophagy in HG-treated NRK-52E cells.

### 3.7. QDD Improved Fibrosis and Autophagy Levels by Inhibiting ATF4 in HG-Treated NRK-52E Cells

**Figure 9:**
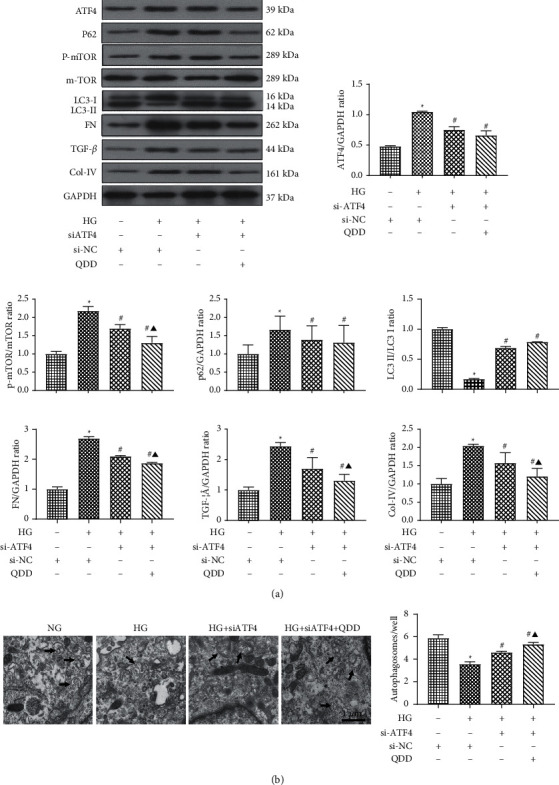
QDD improves autophagy and fibrosis in HG-treated NRK-52E cells by inhibiting the expression of ATF4. (a) Expression and densitometry analysis of ATF4, fibrosis-related proteins FN, TGF-*β*1, and Col-IV, and autophagy-related proteins LC3I, LC3II, p62, and p-mTOR (*n* = 3). (b) Representative electronic micrographs and summarized data showing the number of autophagosomes/well in different groups (autophagosomes were counted in 10 randomly selected fields; scale parameter: 1 *μ*m). Autophagic vacuoles are indicated with arrows. Data are presented as the mean ± SD values. ^*∗*^*P* < 0.05 vs. NG; ^#^*P* < 0.05 vs. HG. ^▲^*P* < 0.05 vs. HG + siATF4. SiATF4: ATF4 small interfering RNA group; Si-NC: siRNA negative control group; NG: low glucose; HG: high glucose.

To further understand the exact mechanism of QDD in alleviating renal tubulointerstitial fibrosis, we transfected NRK-52E cells with siATF4. The protein levels of the autophagy-related proteins P62 and p-mTOR were significantly decreased, and the ratio of LC3-II/LC3-I was obviously increased in the high glucose group transfected with siATF4 compared with the siNC group. Meanwhile, the expression levels of the fibrosis-related proteins FN, TGF-*β*1, and Col-IV were also reduced significantly in the high glucose group transfected with siATF4 compared with the siNC group. Interestingly, compared with the HG group transfected with siATF4, the expression of the p-mTOR protein was significantly downregulated, and the expression of FN, TGF-*β*1, and Col-IV also decreased significantly in the QDD (2000 *μ*g/mL) combined with siATF4 group (Figure 9(a)). In addition, we also detected the level of autophagic vesicles by transmission electron microscopy. Compared with the high glucose group transfected with siNC, the number of autophagic vesicles was obviously increased in the high glucose group transfected with siATF4. Moreover, incubation in HG conditions with 2000 *μ*g/mL QDD combined with transfection of siATF4 significantly enhanced the number of autophagic vesicles compared with that in HG conditions transfected with siATF4 alone (Figure 9(b)). These results suggested that QDD improved the level of fibrosis by inhibiting ATF4 and regulating autophagy in NRK52E cells.

## 4. Discussion

**Figure 10:**
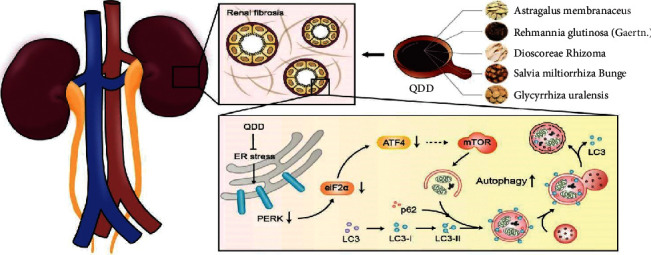
The potential mechanisms of QDD in treatment of DN.

The db/db mouse model of type 2 diabetes has typical pathophysiological characteristics that parallel the development of DN in humans, showing obvious renal tubular interstitial fibrosis [18, 19]. In this study, QDD treatment improved glucose intolerance and alleviated kidney damage and renal tubular interstitial fibrosis in the kidneys of db/db mice. Meanwhile, we found that the PERK/eIF2*α*/ATF4 pathway was activated and the autophagy activity was suppressed in DN mice and HG-treated NRK-52E cells. QDD treatment can enhance autophagy suppression and inhibit the activated PERK/eIF2*α*/ATF4 pathway. Our study provides the first evidence for the outstanding role of QDD in regulating PERK/eIF2*α*/ATF4 pathway-mediated renal autophagy to resist diabetic kidney damage and prevents renal tubulointerstitial fibrosis. The representative cartoon with the potential mechanisms of QDD for DN is shown in Figure 10.

The symptoms related to diabetes are named “Xiao Ke” in traditional Chinese medicine (TCM). According to the theory of TCM, Qi-Yin deficiency with blood-stasis syndrome is an important syndrome of DN. QDD, comprised of *Astragalus membranaceus*, *Salvia miltiorrhiza Bunge*, *Rehmannia glutinosa* (Gaertn), *Dioscoreae rhizoma* and *Glycyrrhiza uralensis*, has the effect of invigorating Qi, nourishing Yin and promoting blood circulation. *Astragalus* has antidiabetic effects [20, 21]. Researchers reported that *Astragalus* polysaccharide upregulates the expression of SIRT1 by inhibiting miR-204, ER stress, and apoptosis in high glucose-induced diabetic retinopathy and metabolic memory models, thereby delaying the development of diabetic retinopathy [22]. At the same time, *Astragalus* polysaccharides can reduce the apoptosis rate of cardiomyocytes by inhibiting the expression of ATF6 and PERK, which are related to the ER stress pathway in diabetic cardiomyopathy rats and high glucose-treated H9C2 cells [23]. *Rehmannia* has the function of nourishing yin and invigorating the kidney in TCM theory and has been widely used for the treatment of diabetes, osteoporosis, and cardiovascular diseases for a long time [24]. Chen et al. found that catalpol, an active ingredient extracted from *Rehmannia*, can reduce podocyte damage in diabetic nephropathy by stabilizing the podocyte skeleton and improving autophagy of damaged podocytes [25]. Rhizoma dioscoreae was first described in the classical medical material book “Shennong Classic Chinese Medicine.” This herb has different pharmacological effects, such as lowering blood sugar and relieving asthma and diarrhea [26–28]. *Salvia miltiorrhiza* can invigorate qi and promote blood circulation. Researchers reported that *Salvia miltiorrhiza* can prevent diabetic nephropathy. The mechanism is not only related to the regulation of the metabolome but may also be related to the inhibition of wnt/*β*-catenin and TGF-*β*1 signaling [29]. In this study, we have identified the potential compounds of QDD, including methylnissolin 3-O-glucoside, trifolirhizin, berrubine, and prazewaquinone A through UPLC-Q/TOF-MS. Methylnissolin 3-O-glucoside is derived from astragalus membranaceus. However, as far as we know, no studies about the effect of methylnissolin 3-O-glucoside have been published yet. Trifolirhizin is a natural flavonoid glycoside, which has the function of anti-inflammatory and regulating autophagy. A previous study confirmed that trifolirhizin can induce autophagy via AMPK/MTOR pathway to prevent and treat colorectal cancer [30]. Berberrubine, a primary metabolite of berberine [31], was demonstrated to have the effect of suppressing the expression of gluconeogenic genes, reducing hepatic gluconeogenesis, and lowering blood glucose [32]. Przewaquinone A is one of the hydroxylated metabolites of tanshinone IIA [33], which has been proven to ameliorate the thickening of the glomerular basement membrane and the collagen deposition in the renal tissues of streptozotocin-induced diabetic rats via attenuating PERK/p-elf2*α*/ATF-4 signaling activities [34]. We confirmed the efficacy of QDD in preventing renal fibrosis in type 2 diabetic db/db mice. However, the mechanism of QDD preventing renal fibrosis and postponing the progression of DN needs further research.

Stress-induced autophagy mainly serves as an adaptive and defense mechanism for cell survival to maintain cell homeostasis [35, 36]. Researchers have confirmed that inhibiting the autophagy of cells in the renal tubules and glomeruli can promote the development of diabetic nephropathy, glomerular interstitial fibrosis and focal segmental glomerulosclerosis [37–39]. Guo et al. found that dihydromyricetin can promote autophagy in NRK-52E cells and reduce renal interstitial fibrosis by regulating miR-155-5p/PTEN signaling and the PI3K/AKT/mTOR signaling pathway in diabetic nephropathy [40]. Zhang et al. found that microRNA-22 accelerates renal tubular interstitial fibrosis by regulating PTEN and inhibiting autophagy in NRK-52E cells in diabetic nephropathy [41]. Our results showed a reduction in the number of autophagosomes in HG-treated NRK-52E cells. Furthermore, qPCR, western blotting, and immunofluorescence indicated that the expression of LC3 was decreased and the expression of p-mTOR was increased in the kidneys of db/db mice. Db/db mice showed critical tubular interstitial fibrosis. Similarly, in HG-treated NRK-52E cells, in addition to the decrease in LC3 expression and increase in p-mTOR expression were observed, the expression of P62 and Col-IV proteins also increased. After QDD treatment, the abnormalities of these indicators were reversed. These results suggested that QDD is of great significance for the treatment of tubulointerstitial fibrosis by regulating autophagy in DN.

Emerging evidences focus on the relationship between endoplasmic reticulum stress (ERS) and autophagy. PERK-eIF2*α* pathway has been reported to participate in the regulation of autophagy [42, 43]. Knockdown of PERK can reduce ERS-mediated autophagy activity and regulation of myocardial fibrosis in H9c2 cardiomyoblasts [44]. A recent study suggested the PERK-eIF2*α* pathway has a critical role in autophagy activation and fibrotic changes during ER stress in renal tubular cells [45]. In our previous study, we confirmed that inhibiting the expression of ATF4 can promote autophagy in NRK-52E cells, thereby reducing tubular interstitial fibrosis in DN [17]. It is increasingly recognized that the activation of endoplasmic reticulum stress (ERS) plays an important role in the development and progression of tubular interstitial fibrosis in DN [46–48]. In the present study, our results showed that the expression of PERK, eIF2*α*, and ATF4 in the kidney tissue of db/db mice was obviously upregulated. To clarify the exact mechanism, we inhibited the expression of ATF4 in NRK-52E cells and transfection of the siATF4 plasmid enhanced autophagy, downregulated the expression of TGF-*β*1, FN, and Col-IV, and improved cell viability. However, of note, ATF4 siRNA only decreased one-third of ATF4 protein levels in NRK-52E cells cultured in HG conditions. Knockdown of ATF4 partly restored autophagy levels in HG-exposed NRK-52E cells. Thus, we hope to explore the effect of QDD combined with siATF4 on autophagy and fibrosis levels in HG-treated NRK-52E cells. Although the combination of QDD and siATF4 did not suppress ATF4 expression markedly compared with siATF4 alone, the combination of QDD and siATF4 has a trend to inhibit the expression of ATF4 more compared with siATF4 alone. We found that intervention with QDD combined with transfection of siATF4 significantly improved autophagy activity and alleviated tubular interstitial fibrosis. We confirmed that QDD can indeed treat renal fibrosis by inhibiting the PERK-eIF2*α*-ATF4 pathway to promote autophagy in diabetic nephropathy.

However, this study still has some limitations. First, although QDD has been analyzed by UPLC-Q/TOF-MS, the effects and potential mechanisms of chemical components of QDD need to be verified in alleviating diabetic renal injuries. Second, this study focused on the relationship between the PERK-eIF2*α*-ATF4 pathway and autophagy, the mechanisms of the PERK-eIF2*α*-ATF4 pathway promote autophagy need to be elucidated in the future. Third, we just evaluated one dose of QDD in vivo and did not explore whether there was a dose-dependent effect of QDD in mice. Therefore, we will further investigate the dose-dependent effect of QDD in diabetic nephropathy mice. Finally, due to the multi-target effect of the Chinese herbal compound, we do not rule out other mechanisms involved in the improvement effect of QDD. We will continue to conduct more research on possible mechanisms in the future.

## 5. Conclusions

This study demonstrated that high glucose upregulated the PERK-eIF2*α*-ATF4 pathway, which in turn suppressed autophagy, resulting in renal fibrosis in DN. QDD can alleviate renal fibrosis by inhibiting the PERK-eIF2*α*-ATF4 pathway and promoting autophagy in DN.

## Figures and Tables

**Table 1:** UHPLC-QTOF-MS data of the top ten characteristic compounds in positive and negative ion modes in QDD.

Ion mode	Compound	Description	m z	Retention time min	Peak area
LQ_POS_ZY	0.64_689.2103 m/z	Stachyose	689.2103345	0.64	20417.82129
	1.74_284.0997 m/z	Guanosine	284.0996836	1.74	14175.53299
	6.91_287.0915 m/z	Licochalcone B	287.0915366	6.91	12850.11229
	8.05_271.0604 m/z	1,3,7-Trihydroxyxanthone1,3,7	271.0604064	8.05	12706.96377
	10.29_311.1276 m/z	Przewaquinone A	311.127614	10.29	10645.50401
	10.13_355.1175 m/z	Gancaonin O	355.1174624	10.13	8781.311916
	11.69_369.1329 m/z	Isoanhydroicaritin	369.1329317	11.69	8437.337111
	10.65_421.3427 m/z	24-Methylene cholesterol	421.3426773	10.65	7804.841081
	5.79_322.1071 m/z	Berberrubine	322.1070792	5.79	7758.648431
	10.68_340.1310n	Blepharolide B	341.1383244	10.68	6606.969026

LQ_NEG_ZY	12.60_371.1841 m/z	Bractealine	371.1840518	12.6	69876.02422
	7.34_507.1486 m/z	Methylnissolin 3-O-glucoside	507.1485894	7.34	23292.95722
	0.92_128.0352 m/z	DL-pyroglutamic AcidDL	128.0351933	0.92	20225.60517
	9.11_991.5100 m/z	Astragaloside VII	991.5100135	9.11	10131.05636
	5.01_491.1165 m/z	Trifolirhizin	491.1165194	5.01	6713.12874
	7.59_464.1660n	Isomucronulator 7-O-glucoside	463.1586828	7.59	3745.445526
	2.75_137.0243 m/z	3,4-Dihydroxybenzaldehyde3,4	137.0242675	2.75	3442.410965
	8.14_271.0598 m/z	Naringenin chalcone	271.0597629	8.14	3325.567429
	6.68_717.1441 m/z	Yunnaneic acid G	717.1441306	6.68	3177.791167
	10.24_331.1536 m/z	Danshenspiroketallactone	331.1535521	10.24	2661.880148

**Table 2:** Quantitative analysis of the key pharmacological components of QDD by UHPLC-QTOF-MS.

UPLC-Q/TOF-MS					
Class	Mzmed	Intensity	Identified name	Composition content (ug/ml)	Formula
NEG	507.1485894	23292.95722	Methylnissolin 3-O-glucoside	0.98080	C_23_H_26_O_10_
NEG	491.1165194	6713.12874	Trifolirhizin	0.28191	C_22_H_22_O_10_
POS	322.1070792	7758.648431	Berberrubine	37.56526	C_19_H_16_NO_4_
POS	311.127614	10645.50401	Przewaquinone A	300.07802	C_19_H_18_O_4_

## Data Availability

The data sets used to support the findings of this study are available from the corresponding author upon request.

## Abbreviations

AMPK: AMP-activated protein kinase
ATF4: Activating transcription factor 4
ATF6: Activating transcription factor 6
BUN: Blood urea nitrogen
DN: Diabetic nephropathy
ERS: Endoplasmic reticulum stress
ESRD: End-stage renal disease
FN: Fibronectin
IRE1: Inositol-requiring 1
LC3: Light chain 3
mTOR: Mechanistic target of rapamycin
NRK-52E: Rat renal tubular epithelial cells
PERK: Protein kinase RNA (PKR)-like ER kinase
QDD: QiDanDiHuang decoction
Scr: Serum creatinine
TCM: Traditional Chinese medicine.
